# G-Quadruplex Matters in Tissue-Specific Tumorigenesis by BRCA1 Deficiency

**DOI:** 10.3390/genes13030391

**Published:** 2022-02-22

**Authors:** Sanghyun Kim, Sohyun Hwang

**Affiliations:** 1 Department of Biomedical Science, College of Life Science, CHA University, Sungnam 13488, Korea; mudol@chauniv.ac.kr; 2 Department of Pathology, CHA Bundang Medical Center, CHA University School of Medicine, Sungnam 13496, Korea

**Keywords:** BRCA1, G-quadruplex (G4), R-loop, tissue-specific-tumorigenesis, basal-like breast cancer, high-grade serous ovarian carcinoma (HGSC), BRCAness, oxidative genome damage, base excision repair

## Abstract

How and why distinct genetic alterations, such as *BRCA1* mutation, promote tumorigenesis in certain tissues, but not others, remain an important issue in cancer research. The underlying mechanisms may reveal tissue-specific therapeutic vulnerabilities. Although the roles of BRCA1, such as DNA damage repair and stalled fork stabilization, obviously contribute to tumor suppression, these ubiquitously important functions cannot explain tissue-specific tumorigenesis by *BRCA1* mutations. Recent advances in our understanding of the cancer genome and fundamental cellular processes on DNA, such as transcription and DNA replication, have provided new insights regarding BRCA1-associated tumorigenesis, suggesting that G-quadruplex (G4) plays a critical role. In this review, we summarize the importance of G4 structures in mutagenesis of the cancer genome and cell type-specific gene regulation, and discuss a recently revealed molecular mechanism of G4/base excision repair (BER)-mediated transcriptional activation. The latter adequately explains the correlation between the accumulation of unresolved transcriptional regulatory G4s and multi-level genomic alterations observed in BRCA1-associated tumors. In summary, tissue-specific tumorigenesis by BRCA1 deficiency can be explained by cell type-specific levels of transcriptional regulatory G4s and the role of BRCA1 in resolving it. This mechanism would provide an integrated understanding of the initiation and development of BRCA1-associated tumors.

## 1. Tissue-Specific Tumor Susceptibility of BRCA1

Mutations in *BRCA1*, which encodes breast cancer type 1 susceptibility protein (BRCA1) [1,2], significantly increase the cancer incidence in several tissues. According to The Cancer Genome Atlas PanCancer Atlas analysis on oncogenic molecular processes, heterozygous carriers with germline *BRCA1/2* mutations develop cancer at younger ages compared to the wild-type in ovarian serous cystadenocarcinoma, lung squamous cell carcinoma, and breast invasive carcinoma [3]. The previously best-known tissues exhibiting cancer susceptibility of BRCA1 are the breasts and ovaries [4]. *BRCA1*-mutant tumors in the breast tend to exhibit a basal-like phenotype and often have a triple-negative breast cancer (TNBC) phenotype that lacks the expression of estrogen receptor, progesterone receptor, and human epidermal growth factor receptor 2 [5,6]. In ovarian tissue, *BRCA1*-mutant tumors are mostly high-grade serous carcinoma (HGSC) [5,6]. Despite differences between these tissues, these two best-known BRCA1-deficient tumors share many molecular properties [7,8] including mutations in the tumor suppressor gene *TP53*, the amplification of the *MYC* oncogene, extreme levels of genomic instability and copy number variation (CNV), and sensitivity to DNA damage agents. These properties are termed BRCAness [5,6]. However, BRCA1 deficiency is not always associated with BRCAness, as *BRCA1* alterations in non-BRCAness cancer seem neither related to tumor pathogens nor therapeutically actionable [9,10]. 

This tissue-specific tumorigenesis has been a critical question from the beginning of BRCA1 research [11], in that the mechanism underlying tissue-specificity of cancer-associated molecular alteration may also reveal tissue-specific therapeutic vulnerabilities and preventive strategies. Cancer driver genes that are expressed in a wide variety of tissues, not restricted to tissues from which the cancer originates, could contribute to tissue-specific tumorigenesis through (i) tissue-specific oncogenic functions of cancer drivers, and (ii) the characteristics of the cell-of-origin, that is, tissue context [12]. In terms of molecular function, the tissue-specific function of BRCA1 has not yet been reported. The roles of BRCA1 in genetic stability, such as homologous recombination-based double-strand break (HR-DSB) repair and replication fork stabilization [13,14], are known to contribute to tumor suppression. However, these ubiquitously important functions per se cannot account for tissue-specific tumorigenesis. In addition, tumorigenic effects of *BRCA1* mutation are strongly associated with somatic biallelic inactivation. However, haploinsufficiency may also promote the formation and progression of tumors [6,15], and the rate of biallelic inactivation of *BRCA1* in pathogenic germline carriers is cancer type-specific [9]. Therefore, tissue context has become an inevitable and natural consideration in BRCA1-associated tumors and throughout in cancer biology [12,16]. Tissue-specificity exists not only in tumorigenesis, but also in the therapeutic action of common molecular alterations shared between different tumor types. Recent basket trials using off-label targeted drugs in patients with the same genomic alterations but with different cancer types have provided evidence that the response to a molecular alteration-specific anticancer drug often depends on the anatomical cancer type [12]. Tissue context can be described as follows: different cell types have different epigenetic states dictating which genes are expressed and which genes are potential to be activated in response to stimuli, and thereby have different epi-proteome states determining which signals are capable to be sensed and in what manner a cell can respond [12,16]. This includes environmental factors as well.

A recent review of tissue-specific tumorigenesis by *BRCA1/2* mutations summarized aspects of this tissue context [17]. The explanation given for tissue-specific tumorigenesis by BRCA1 deficiency thus far is that repetitive exposure to estrogen causes a greater need for HR-DSB repair [18], or that BRCA1 loss of function can only be tolerated in these tissues via estrogen-induced pathway response [19]. These hypotheses are relevant only for BRCAness tumors originating in hormone-responsive tissues. A more recent suggestion is that tissue-dependent tumor-suppression of BRCA1 may be associated with its roles in transcriptional and epigenetic regulation [20]. However, these arguments still remain phenomenological and do not provide a unified explanation at the molecular level [16].

Recent advances in our understanding of the cancer genome and fundamental cellular processes on DNA at molecular levels, such as transcription, replication, and 3D genomic organization, have provided new insights into BRCA1-deficient tumorigenesis. These advances have allowed us to better understand the interrelationships between molecular processes occurring on DNA and cancer mutational landscapes. Collectively, these findings suggest that one of DNA secondary structures, G-quadruplexes (G4s), plays a critical role. In addition to the general importance of the G4 structure, G4 exhibits significant relevance to BRCA-deficient tumors, in that G4-stabilizing ligands are synthetic lethal with BRCA1/2 deficiency [21,22,23,24,25]. Herein, we review recent studies on the importance of G4s in the mutational landscapes of the cancer genome and in cell-type-specific transcriptional regulation, and summarize the evidence that the accumulation of unresolved G4s is responsible for tissue-specific tumorigenesis by BRCA1 deficiency.

## 2. G-Quadruplex

### 2.1. Introduction to the G-Quadruplex

**Figure 1:**
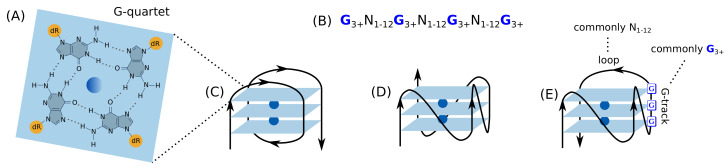
The structure and topologies of G4. (**A**) Structure of a G-quartet formed by hydrogen bonded four guanines and central cation (blue). (**B**) The consensus sequence of G4. Representative topologies of unimolecular G4s based on the strand direction: (**C**) antiparallel, (**D**) parallel, and (**E**) hybrid.

G4s are non-B DNA secondary structures of stacked guanine (G) quartets, in which four guanine molecules form a square planar arrangement via hydrogen bonds (Figure 1). These structures have vast structural diversity depending on the length and the constituent bases of the loops, the constituency and the direction of the strands, and the specific G-tracks involved in G4 formation if there are more than four G-tracks. 

DNA exist predominantly in the form of a double helix B-conformation in cells, and the G4 structure was previously considered to be adopted by DNA only *in vitro* [26]. However, the G4 structure began to attract attention when it was reported that the human telomeric DNA sequences form the G4 structure [27]. In the same year, it was directly demonstrated that this structure exists in the promoter of the representative oncogene, *c-MYC*, and *c-MYC* expression is down-regulated by a G4 stabilizing small ligand [28]. Numerous studies have since investigated the G4 structure in the *MYC* promoter region and its interaction with small molecules to regulate *MYC* expression [29,30,31].

The genome-wide prevalence of G4 has been demonstrated. Bioinformatics studies have revealed that potential G4 forming sequences (PQSs) are prevalent in the human genome [32,33], particularly in regulatory regions [34], including in the promoter region of various oncogenes such *as c-Kit, KRAS, VEGF-A, Bcl-2*, and *Hif-1α* [35]. These findings were reproduced and strengthened in the experimental detection of G4s [36,37]. In an *in vitro* experiment, 716,310 G4s were identified, of which more than 60% were not predicted by the bioinformatics method [36]. This also showed a significant association between G4 and oncogenes, tumor suppressors, and somatic CNVs [36]. In an endogenous chromatin context, chromatin immunoprecipitation and next-generation sequencing (ChIP-seq) of G4 resulted in the detection of approximately 10,000 G4s, predominantly in regulatory nucleosome-depleted regions associated with genes showing elevated transcription [37]. An enrichment of G4s in cancer-related genes and/or somatic CNVs was also re-demonstrated. *MYC* showed the highest G4 ChIP signal among all cancer-related somatic CNV amplification and oncogenes [37]. These results support a previously suggested role [38] for G4 structures in tumor progression. Moreover, the fact that G4s are particularly enriched in somatic CNVs [36,37] draws attention to the relevance of G4s in BRCA1-deficient tumors in that these tumors display high levels of CNVs [8,39].

The single-molecule visualization of G4s either *in vitro* or in live cells revealed that G4s dynamically fluctuate between folded and unfolded states at a time scale of a few seconds. Their formation was found to be associated with the active processing of DNA, that is, replication and transcription [40]. Currently, G4s have become an emerging therapeutic target in oncology [38,41,42,43,44] because of its significant association with oncogenes, its potential for transcriptional regulation, and its structural diversity suggestive of selectivity. General reviews of G4 can be found in recent articles [45,46,47].

### 2.2. G4s as the Determinants of Mutagenesis

G4s are strongly associated with mutagenesis and CNVs. Even before demonstration of genome-wide enrichment of G4s in somatic CNVs [36,37], the correlation between non-B DNA structures, including G4s, and CNVs was first reported through a direct experiment by the transformation of the engineered plasmid into *E. coli* and induction of a report gene expression [48]. A 2.5 kilobase sequence known to form non-B DNA structures was found to increase the frequency of plasmid alterations, including long deletions, in conjunction with DNA repair processes and transcription. 

The crucial contribution of G4 to mutational landscapes has been revealed by large-scale whole genome sequencing (WGS) analysis [49,50]. It was previously known that mutation rates in the human genome depend on megabase-sized characteristics such as replication timing and chromatin organization [51,52]. However, local features influencing mutation rates, such as non-B structure motifs, have only recently been identified through an enrichment analysis at short genomic length scale, in combination with regression models predicting mutation rates [49,50]. Density analysis of non-B structure forming motifs at a small window size of 2-kilobases using WGS of 1,809 patients from 10 cancer types clearly showed their enrichment at sites of somatic mutation [49]. Furthermore, the increased mutability associated to non-B motif depends on the physical formation of secondary structures. That is, the elevated mutation densities are domain-specific within the non-B DNA structures and associated with biophysical characteristics such as a loop length. For example, within inverted repeats that tend to form hairpins or crosses, loop sequences exhibit more abundant substitutions than stem sequences. G4s have about 1.15 to 1.8 times higher mutation frequency in loops than in G-tracks. G4s with average loop size of up to 3 nucleotides are more mutable than those with larger loops [49]. All these statistical analyses support that non-B secondary structures were not simply associated with increased mutation density but were causally implicated [49]. 

Similar results were reproduced in an independent study using deep whole genome sequencing data from 300 individuals [50]. Even for large-scale regional variations in the frequency of nucleotide substitutions at a 1-megabase window, non-B DNA structures could explain more variation than any other predictors such as replication timing, histone marking and distance to telomeres in multiple regression models. This showed that loci capable of forming non-B DNA structures are a major driver of variation in nucleotide substitution levels across the genome, at both small and large scales [50]. 

This significant contribution of G4s in mutation rates can also be attributed to the fact that G4s exhibit a strong association with other large-scale factors influencing the mutational landscape. For example, G4 structures are involved in functions of key architectural proteins such as CTCF [53] and YY1 [54]. CTCF is known to cluster at the boundaries of topologically associating domains (TADs) [53,55], and also mediates enhancer-promoter interactions [56,57,58] through promoter-proximal binding [59,60]. In general, chromatin organization, in which architectural proteins play a key regulatory role, has a major influence on regional mutation rates in human cancer cells [61]. Indeed, CTCF binding sites are frequently mutated in cancers, including colon cancer, stomach cancer, and melanoma [62,63,64,65,66]. G4s influence the regional variations of mutation rates associated with CTCF, in that CTCF are preferentially located surrounding G4 which can strengthen the insulation ability of CTCF binding sites [67]. An *in vitro* assay demonstrated that G4s contribute to CTCF recruitment [68]. Another structural regulator of enhancer-promoter loops, YY1 [54], is also a DNA G4-binding protein, and YY1-mediated long-range DNA looping occurs through its recognition of the G4 structure [69].

G4 structures are also associated with replication timing, the first recognized genomic feature related to the mutation rate in humans [70]. The DNA replication origins are preferentially associated with an origin G-rich repeated element that potentially forms G4s, and G4s are functionally important in replication initiation [71,72]. It is generally accepted that chromatin organization and replication timing shape the mutational landscape of cancer together [61,73,74], and are in fact associated with each other [75]. Recently super-resolution imaging has shown that the spatiotemporal propagation of human replication foci is mediated by CTCF-organized chromatin structures [76]. All these results suggest the importance of G4s in the mutational landscape of the cancer genome.

The most correlated genetic structural element with G4s is a R-loop, a three-stranded nucleic acid structure containing a DNA-RNA hybrid duplex and a displaced single DNA strand. This structure has received great attention due to growing evidence that it is essential for gene regulation and DNA repair, despite being considered previously a toxic by-product of transcription, triggering genomic instability. Such dual effects of R-loop, as cellular regulators and genomic threats, have been extensively discussed in other reviews [77,78,79]. The primary evidence for an association between R-loop and G4 was provided by a study on ChIP-seq of R-loop (DRIP-seq), and G4. DRIP-seq peaks show a prevalence at the 5’ and 3’ ends of GC-skewed, transcriptionally active loci [80,81,82], similar to G4 ChIP-seq. More recent reports have shown that the formation of a G4 in one strand is highly favored by a DNA:RNA hybrid duplex in the opposite strand, and vice versa [83,84,85]. This characteristic points to a novel structure, termed G-loop, containing a G4 on one strand and an R-loop on the other, which is transcription-dependent [86]. 

In some cases, R-loop formation precedes and facilitates G4 formation [84,85]. In other cases, G4 folding in the promoter region is independent of transcriptional activity, thereby preceding transcription [87]. Although the detailed mechanism of G-loop formation has yet to be fully elucidated, it is clear that R-loops and G4s are intertwined and can promote each other during transcription. Their interplay during transcription may be position- or context-dependent in that the terminal R-loop formation, in contrast to the promoter-proximal R-loop, is not as highly associated with GC skew, thereby implying a different mechanism of G-loop formation at each gene end [82]. It is likely that in the promoter-proximal region with a high GC content, G4 folding precedes and facilitates R-loops, whereas in the terminal region, R-loops promote G4 formation. The interrelation between G4s and R-loops is not limited to transcription, but also exists when causing DNA damage [88]. G4 ligand-induced DNA damage and genome instability are mediated by R loops. The close structural interplay between G4s and R-loops has been well documented elsewhere [89].

### 2.3. G4s Are Key Genomic Structural Elements in Transcriptional Regulation

From the beginning of G4 research in the early 2000s, the regulatory potential of G4s on transcriptional activity has been an important motivation. The role of G4s in transcriptional regulation has been strongly supported by reports on the down regulation of individual oncogenes (e.g., *MYC* [28] and *H-RAS* [90]) by G4 stabilizing ligands (i.e., pyridostatin). The genome-wide enrichment of G4s in the promoter region [36,37,91] also supports this hypothesis. The single-molecule visualization of G4s revealed that dynamic G4 formation is associated with transcription, as G4 dynamics are disrupted by the inhibition of transcription [40]. The same conclusion was derived by a genome-wide interaction study between PQS and single nucleotide variation (SNV) and their impact on transcription [92]. SNV in PQS can impact the G4 structure, thereby resulting in G4 variation (G4V). The majority of G4Vs overlap with gene regulatory elements, such as transcription factor (TF) binding sites and enhancers, and G4Vs in the regulatory regions have been reported to exhibit a significant influence on gene expression [92]. 

A growing number of studies even suggest that the G4 structure may be sufficient to direct cell-type specific transcription, rather than simply involved in transcription. While two cell lines have different G4-folding states in the same loci, high transcript levels are consistently associated with G4-folding, and TF binding sites are highly enriched within G4 ChIP peaks [93]. This study concluded that promoter G4s and TFs cooperate to determine cell-specific transcriptional programs [93]. A similar result was observed independently by S. Balasubramanian’s group [87,94]. G4s operate as common binding hubs for many different TFs to promote transcriptional output [94]. Furthermore, they provided evidence that promoter G4 folding precedes transcription by showing that promoter G4 formation does not depend on transcriptional activity; the transcriptional inhibition of the catalytic subunit of PTEF-b did not cause statistically significant changes in the promoter G4 ChIP signal at most of sites [87]. 

A recently revealed molecular mechanism of oxidative DNA damage-mediated transcriptional activation provides an explanation for the transcriptional regulatory role of G4 [95,96]. This is congruent with the previously known mechanism in which generation of 8-oxo-7,8-dihydroguanine (8-OxoG) in a promoter region and a subsequent base excision repair (BER) process are the essential initiating steps for transcription activation [97,98,99]. These regulatory processes have been observed for various transcriptional activation, including NF-κB (nuclear factor kappa B) activation [100,101], and estrogen- [102], hypoxia- [103], Myc- [104,105], and retinoic acid-induced transcription [106]. 

8-OxoG is generated site-specifically in the promoter regions of specific genes, because the local reactive oxygen species (ROS) are generated by histone demethylation during transcription initiation [98,107]. The oxidation of DNA by ROS mostly drives guanine to 8-OxoG conversion because G-tracks have the highest oxidation propensity and the duplex DNA can funnel electron holes to oxidation-susceptible sites [108]. When the target promoter is marked with oxidized guanine, the BER of this 8-OxoG is initiated by 8-Oxoguanine glycosylase I (OGG1), which cleaves 8-OxoG to generate an abasic (apurinic/apyrimidinic, AP) site. Transcription induction was found to require OGG1 and apurinic/apyrimidinic endoDNase I (APE1) [97,98,99]. In this context, the recent studies provided a mechanistic link between G4 formation/stabilization and the repair process of oxidized DNA in transcriptional activation [95,96]. 

**Figure 2:**
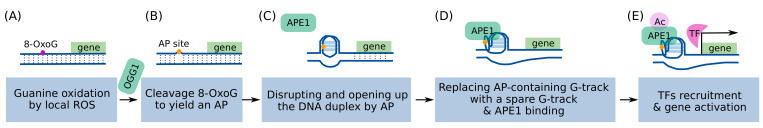
Proposed mechanism of G4/BER-mediated transcriptional activation. (**A**) Local ROS generates 8-OxoG in the G-rich regions of the promoter, (**B**) which is removed by OGG1 to form an AP site. (**C**) The AP site rearranges the DNA duplex into a G4 structure, and (**D**) a more stable G4 can be formed by involving the fifth G track and looping out the AP site. (**E**) APE1 can bind the AP site and recruit TFs.

The suggested model encompasses sequential molecular events (Figure 2) [95,96]: local ROS generate 8-OxoGs from a promoter PQS; OGG1 cleaves the 8-OxoG to generate an AP site; the AP site opens the DNA duplex and allows a G4 to fold; the G-Track having AP site is replaced with a spare G-track, and the G4 structure is stabilized; APE1 binds to the exposed AP site and recruits TFs. Gene activation is known to occur via the redox effector function of APE1, which increases the DNA binding activity of TFs by modulating the redox status of reactive Cys residues in the DNA-binding domain of TFs [109]. Many TFs, such as NF-κB, AP-1, CREB, Egr-1, HIF-1a, and p53, are known to have their DNA-binding activity regulated by this redox mechanism [109].

Transcription by this mechanism strongly depends on the positional context of the promoter G4s. According to a quantitative evaluation of expression levels using the synthetic promoter PQS, transcription is either up- or down-regulated depending on the location and the strand in which 8-OxoG or its following AP resides [110,111]. The presence of G4 on the non-template strand was found to result in a higher expression level. Such increased transcription in the presence of G4 on the non-template strand is due to the formation of an R-loop on the opposite strand, that is, the formation of a G-loop [85].

In this genomic structural context of G4/BER-mediated transcription, expression levels can be modulated by several factors. Representatively, the acetylation/deacetylation of APE1 plays an important regulatory role, modulating the residence time of APE1 on the G4 structure, thereby modulating transcription levels [96]. Therefore, p300 and SIRT1 are important regulatory factors in G4/BER-mediated transcription, since p300 acetylates APE1 and SIRT1 deacetylates APE1 and promotes its dissociation from the G4 structure [112,113].

This mechanism is expected to be prevalent in various respects. First, the same mechanism was demonstrated in the transcriptional regulation of other genes such as *NTHL1* [95], *KRAS* [114], *RAD*17 [115], and *PCNA* [116]. The expression of *APE1* and *SIRT1* is also regulated by the G4-BER mediated mechanism [117,118]. In addition, previous reports regarding transcriptional regulation by 8-OxoG and BER factors have already accumulated for various transcription factor activities with a wide range of downstream transcription activations [97,98,99]. The genome-wide profiling of G4s and the binding loci of BER factors upon APE1 knockdown provides further evidence for the prevalence of this mechanism [96]. Binding locations of G4-specific antibody and acetylated APE1 (acAPE1) and OGG1 (acOGG1) significantly overlapped with differentially expressed genes following APE1 knockdown [96]. On the other hand, genome-wide statistics of promoter PQS are also noteworthy for estimating the prevalence of the mechanism. Among human gene promoters, 42.7% contain PQSs [34], and more than 40% of all PQSs have five or more G tracks [119]. Although fine details may differ, the overall mechanism of transcriptional activation, which includes guanine oxidation in the promoter PQS and BER recruitment accompanied by replacement of an AP carrying G-track with an extra G-track, appear to be basically the same. Several reviews have discussed the interplay between guanine oxidation and promoter G4 folding for transcriptional regulation [108,120,121].

## 3. The Role of BRCA1 in Resolving Regulatory G4s That Can Induce DNA Damage

### 3.1. Increased Levels of Transcriptional Regulatory G4s Can Cause DNA Damage

In the previous section, we summarized the role of G4s as a determinant of mutagenesis and a key genetic structural element for transcriptional regulation. Moreover, G4s are significantly enriched in somatic CNVs [36,37]. This leads to speculation of a causal link between tissue-specific transcriptional activity and genomic alterations, particularly CNVs, in a G4-dependent manner. Indeed, several studies have reported significant correlations between distinct transcriptional activity and somatic CNVs. For example, a study screened proliferation regulators in multiple cell types and investigated whether they were associated with recurrent focal regions of CNVs and aneuploidy patterns [122]. The profiled proliferation drivers exhibited striking cell-type dependence and specific enrichment in somatic CNVs of cognate tumors. These cell-type-specific proliferation drivers helped predict tissue-specific aneuploidy patterns. This suggests that the tissue specificity in proliferation-driving transcriptional control underlies somatic CNVs and CNV-associated cancer driver selection in different cancers [122]. 

Furthermore, a comparative analysis of G4 ChIP-seq on 22 breast cancer patient-derived tumor xenograft (PDTX) models suggests that the correlation between the transcriptional program and CNVs arise in a G4 dependent manner [123]. This study profiled differentially enriched G4 forming regions (ΔG4Rs) in each PDTX, and investigated overlaps of CNVs and ΔG4R enrichment in the binding sites of 134 TFs. As expected, ΔG4Rs showed significantly enrichment in regions of CNV and SNV, as well as in the promoter regions of highly expressed genes. ΔG4R fold enrichment in TF binding sites across PDTX models revealed distinct TF programs that were differentially active across the PDTXs [123].

The causal relationship between G4s and genomic alterations has been well known by the previous studies. G4s act as obstacles during DNA replication causing the stalling and collapse of replication forks [124,125,126]. Double strand break (DSB) and broken forks are the source of CNVs by non-HR repair mechanism [127]. Genomic instability caused by G4s has been reported mainly in relation to the function of various helicases such as Pif1 [128] and FANCJ [129], and has been well discussed in recent reviews [124,130]. However, since the transcriptional regulatory role of G4 was only recently elucidated, it was not recognized that G4-associated DNA damage could be associated with cell-type-specific transcriptional activity.

The first observation of the correlation among G4 and transcriptional alteration and DNA damage was already made ten years ago through the genome-wide mapping of damaged genes by the G4-stabilizing ligand, pyridostatin [131]. Cells treated with pyridostatin exhibited transcription-and replication-dependent DNA damages, and ChIP-seq analysis of the DNA damage marker ɣH2AX showed that pyridostatin targets genes containing PQS clusters. Parallel expression profiling showed that ɣH2AX-positive genes showed significantly altered gene expression compared to the ɣH2AX-negative control genes. This indicates a strong correlation among the alteration of the G4 dynamic structure and transcriptional levels and DNA damage at specific gene loci [131]. The same result was demonstrated using other G4 ligands, and DRIP-seq analysis further showed that R-loops are involved in the interplay between DNA damage and transcriptional alteration in the promoter G4-containing genes [88]. 

A correlation between DNA damages and co-transcriptional R-loops or G4s (most probably G-loop) was also observed in hormone-induced transcription activation in breast cancer cell lines treated with estrogen (E2, 17ß-estradiol) [132]. E2 induces DSBs in a replication- and transcription-dependent manner. DRIP-seq analysis revealed that estrogen exposure causes a rapid, global increase in R-loop formation in a transcription-dependent manner, and genomic rearrangements are enriched in E2-responsive genes. E2-induced R-loops were colocalized with DNA damage markers on chromatin and ribonuclease H (RNase H) resolving R-loop reduced E2-induced DNA damage [132]. These results indicate that replication-dependent E2-induced DNA damage results from these co-transcriptional R-loops. Here, the co-transcriptional R-loops are actually G-loops. As mentioned before, co-transcriptional R-loops are highly favored by G4s in the opposite strand [83,84,85], and this study confirmed a significant overlap between the DRIP-seq peaks and the previously profiled G4-forming regions [132]. 

All these indicate that gene-expression-related genomic stress, G4s or G-loops, is cell-type specific and is responsible for DNA damage followed by CNVs.

### 3.2. The Accumulation of Transcriptional Regulatory G4s and DNA Damage Depends on the BRCA1 Status and This Dependency Is Cell-Type Specific

How much of this transcription regulatory G4/R-loop is generated in basal level is cell-type specific [133]. DRIP-seq analysis of distinct cell types of fresh normal breast tissue showed that R-loops are more pronounced in luminal cell populations than in basal and stromal cells, at transcription start sites and termination sites [133]. In addition, this cell-type specific R-loop accumulation was more severe in BRCA1 mutation carriers than in non-carriers. This finding implies that gene-expression-related genomic stress is higher in BRCA1 mutation carriers and only in a certain cell-type such as luminal cells in breast tissue. Although the positional overlap between R-loop and G4 was not evaluated in that study, the observed DRIP peaks had a high GC skew, which is highly probable for G-loop formation [133]. This result strongly suggests that BRCA1 is involved in regulating R-loops/G4s and suppressing their accumulation.

The tumorigenic luminal-specific G-loop accumulation in breast tissue is linked to RANK/RANKL-induced transcriptional activation for two reasons. First, TNBC originates from luminal epithelial progenitors (LPs) [134,135,136], and the pathway of receptor activator of NF-κB (RANK) and its ligand (RANKL) in LP cells is a critical contributor of TNBC tumorigenesis [137,138,139,140]. In addition, RANK expression is luminal-specific, and RANK+ LP cell fraction is much higher in BRCA1 mutation carriers than in BRCA1 wild-type individuals [139]. Indeed, it has been shown that NF-κB is persistently and autonomously activated in a subset of BRCA1-deficient mammary luminal progenitors which drives aberrant proliferation and an accumulation of DNA damage [141]. Second, as mentioned above, transcription induced by NF-κB activation occurs via the G4/BER-mediated mechanism [100,101]. 

In summary, certain transcriptional activities, such as NF-κB activation, produce higher levels of transcriptional regulatory G4s, and consequently, a higher demand for G4 processing makes the BRCA1 status important for maintaining G4 levels and preventing DNA damage. However, it has not been determined whether R-loop/G4 level is higher in *BRCA1*-mutation carriers by E2-induced transcription, which also causes a large amount of G4/R-loop. It may be so, since it is known that E2-induced gene expression is inhibited by BRCA1 overexpression [142,143] in a p300-regulated manner [144].

## 4. Consequence of BRCA1 Haploinsufficiency

### 4.1. BRCA1 Heterozygosity Cause a Cell-Type Specific Haploinsufficiency for Resolving G4s

BRCA1 involvement in the link between DNA damage and G4 accumulation was also revealed by a study that evaluated the functional sufficiency of heterozygous BRCA1 in histologically normal mammary tissue [145]. This was performed to identify a driving factor to initiate the mammary tumorigenic process, as inherited mutations in *BRCA1* are known to cause specific molecular and cellular alterations in breast tissue even before cancer development [146,147,148,149]. *BRCA1* mut/+ retained normal functions of centrosome number control, spindle pole formation, and satellite RNA suppression [145]. In addition, there was no significant difference between *BRCA1* +/+ and mut/+ for DNA damage checkpoints, when assessing the proportion of DNA-synthesizing cells after UV-induced DNA damage. The same result was observed for DNA repair function, assessed by RAD51 recruitment as an indicator of a key step in HR and sensitivity measurement to PARP inhibitors. However, in the presence of replicating-stalling agents such as hydroxyurea (HU) or UV radiation, *BRCA1* mut/+ exhibited inefficient recruitment of phospho-RPA32 on chromatin, an abnormally high frequency of collapsed forks, and increased degradation of the nascent replicating strand. Furthermore, in the presence of sufficient replication stress, HR-DSB repair was also defective in *BRCA1* mut/+ cells. This is known as “conditional haploinsufficiency” of *BRCA1* mut/+ for HR-DSB repair, wherein the pool of BRCA1 available for previously intact functions is reduced [145]. The limited quantity of BRCA1 may induce innate or conditional haploinsufficiency depending on the biological context or environmental stimulus. 

However, deficiency in SFR may not be simply a deficiency in SFR itself, but a consequence of defective G4 resolution. When evaluating BRCA1 functional sufficiency, an assessment for SFR was performed using the common replicating-stalling agents, HU and/or UV [145]. Although HU is mostly known to deplete nucleotide pools by inhibiting ribonucleotide reductase, which catalyzes the rate-limiting step in the biosynthesis of dNTP precursors [150], a recent report suggested that HU not only depletes nucleotides, but also induces G4 formation, followed by G4-dependent DNA damage, heterochromatin formation, and perturbed gene expression [151]. Across the genome, chronic exposure to HU results in an altered pattern of gene expression similar to that seen in cells lacking the G4-unwinding helicases FANCJ, WRN, and BLM. The affected genes were enriched in the G4 motifs [151]. In addition, when assessing the functional sufficiency of heterozygous BRCA1 [145], global G4 accumulation and alterations in gene expression observed in normal mammary epithelial cells from BRCA1-mutation carriers compared to wild-type carriers [147,149], were overlooked. Therefore, G4 resolution may be the first defective function of BRCA1 haploinsufficiency, and BRCA1 haploinsufficiency for SFR may be a conditional insufficiency after the accumulation of unresolved G4s.

### 4.2. Altered Gene Expression Caused by BRCA1 Haploinsufficiency Can Lead to Cell-Type-Specific Genomic Instability and Premature Senescence

Interestingly, rather than BRCA1 haploinsufficiency for SFR, haploinsufficiency for G4 resolution causing altered gene expression seems to result in cell-type-specific genomic instability [152]. Once G4s accumulate, haploinsufficiency for SFR is not limited to a certain cell type, as observed in both fibroblasts and epithelial cells [145]. However, G4/R-loop accumulation by BRCA1 haploinsufficiency and consequent alterations in gene expression are cell-type-specific [133]. Among genes whose expression is altered by misregulated G4s, key phenotypic regulators may be included [152]. BRCA1 haploinsufficiency leading cell type-specific genomic instability and phenotype was examined in the primary cells of disease-free breast and skin tissue from either BRCA1 mutant or wild-type carriers [152]. Prolonged passage of BRCA1 heterozygous cells showed cell type-specific phenotypes. Human mammary epithelial cells from *BRCA1* mut/+ have been reported to exhibit increased genomic instability, rapid telomere erosion, and premature BRCA1 haploinsufficiency-induced senescence. Primary keratinocytes showed premature senescence, but were not associated with telomere dysfunction. Fibroblasts, either from the human mammary or dermis, did not exhibit premature senescence [152]. 

This cell-type-specific phenotype caused by BRCA1 haploinsufficiency was found to be related to NAD+ dependent deacetylase SIRT1 at molecular level [152]. A decrease in the SIRT1 levels leads to the accumulation of acetylated H4K16 (histone H4 on lysine 16) and acetylated pRb, thereby resulting in telomere erosion, genomic instability, and pRb-dependent premature senescence. This implies that the phenotype with premature senescence and telomere erosion in the long-term culture of *BRCA1* mut/+ cells is associated with the misregulation of SIRT1 by BRCA1 haploinsufficiency [152]. *SIRT1* is one of the many genes altered by BRCA1 haploinsufficiency [147,149]. This protein-deacetylase is involved in various cellular processes [153,154] including DNA damage repair and telomere maintenance [155,156,157]. In addition, SIRT1 is a critical modulator of G4/BER-mediated transcription by deacetylating APE1 [112,113], and its own expression is regulated by a G4/BER-mediated mechanism [117,118]. Therefore, SIRT1 is a significant feedback factor that affects G4/BER-mediated transcriptional regulation and phenotype. SIRT1 is known to have an important and unique association with BRCAness tumors. Its expression level has been reported to increase in a number of tumor types [153,158]; however, some cancers, such as breast and ovarian, show down-regulated levels of SIRT1 [159,160]. The effects of SIRT1 on promoting senescence or negatively regulating its own expression in cells are known to depend on the presence or absence of p53 [153]. It has yet to be elucidated whether SIRT1 down regulation and a high frequency of *TP53* mutations in BRCA1-associated tumors are correlated. However, it has been clearly demonstrated that BRCA1-deficient breast cancers have lower levels of SIRT1 than the corresponding normal controls, and the ectopic expression of SIRT1 has been reported to inhibit *BRCA1* mutant cell growth and tumor formation in a mouse model, but not in the BRCA1 wild-type [159]. 

In addition to SIRT1, BRCA1 deficiency alters the expression of many other factors that can affect G4/BER-mediated transcription, such as NRF2, CYP1A1, RANKL, OGG1, and APE1. Primary mammary epithelial cells from BRCA1-deficient mice show low levels of Nrf2 expression, a master regulator of the cellular antioxidant response, and Nrf2-transcriptional targeted antioxidant enzymes [161]. They determine the ROS levels and redox status in cells which influence the oxidation of guanine and G4 folding. BRCA1 also regulates estrogen metabolism-mediated DSB by repressing the transcription of estrogen-metabolizing enzymes such as CYP1A1 in breast cells [162]. Regardless of the estrogen receptor status, estrogen release can cause damage and genomic instability via catechol estrogen metabolites [163,164]. Tissue-specific conversion to catechol estrogen metabolites, along with the subsequent formation of ROS and unstable catechol estrogen intermediates, was one of the early explanations for tissue-specific tumorigenesis due to BRCA1 deficiency in estrogen responsive tissue [163]. In addition, BRCA1 haploinsufficiency upregulates RANKL expression and cell proliferation [165], which contributes significantly to TNBC tumorigenesis from the cell-of-origin [137,138], and RANKL inhibition markedly attenuates tumor onset [139,140]. BRCA1 also regulates the transcription of major BER enzymes, such as OGG1 and APE1 [166]. 

It is not yet known whether altered expression of these genes by BRCA1 haploinsufficiency is cell type-specific and whether such an alteration in the expression of a certain gene contributes to a cell-type-specific phenotype. However, these suggest that defects in resolving transcriptional regulatory G4s cause transcriptional alterations in many genes, among which a certain gene, such as *SIRT1* [155,156,157], may be associated with a cell type-specific phenotype as a context-dependent cancer driver. 

### 4.3. BRCA1 Insufficiency Causes Multi-Level Heterogeneous Molecular and Cellular Alterations

BRCA1 heterozygosity can cause sequential conditional haploinsufficiency in the following three distinct functions of BRCA1. First, BRCA1 haploinsufficiency for processing G4s may result in altered gene expression even before malignant tumor onset in BRCA1 mutation carriers [146,147]. Second, if BRCA1 deficiency becomes more severe, stalled replication forks will accumulate and repair may not be sufficient [145]. Third, once BRCA1 haploinsufficiency results in defective SFR, the impaired stalled forks can leave more deleterious DSBs [167] and CNVs by non-HR repair mechanism [127]. That is, a severe accumulation of G4s can induce conditional haploinsufficiency for SFR or HR-DSB repair sequentially leading to genomic instability. In addition to the conditional haploinsufficiency for SFR and HR-DSB, the altered expression of certain genes, such as *SIRT1* [155,156,157], further contributes to genomic instability. 

In addition to transcriptional alteration and genomic damage, the dysregulation of the transcriptional regulatory G4s can cause epigenetic alterations, including changes of histone marks and DNA methylation pattern. First, poor G4 processing during replication leads to epigenetic instability in which epigenetic chromatin marks are not well transmitted to daughter cells (see [168] and references therein). This is because the DNA helicase and polymerase are uncoupled as the helicase continues to unwind the parental duplex, even when the leading strand polymerase encounters a persistent G4 structure and is blocked. Delayed replication of excessive single strand DNAs between the helicase and the polymerase results in loss of parental histones that can be recycled during reestablishment of chromatin. Parental histone recycling is important for maintaining the parental expression status by propagating parental histone marks to newly formed chromatin after replication [168]. 

In the other hand, DNA G4 structures mold the DNA methylome by sequestering DNMT1 and locally inhibiting methylation at specific CpG islands [169]. In addition to regulating DNMT1 transcription [170], this means that BRCA1 influences location-specific, genome-wide methylation landscape by regulating G4s. This may account for the epigenetic alterations such as a lower methylation level in CpG island promoters, observed in BRCA1 deficient tumors [170,171,172,173]. Furthermore, the fact that unresolved G4s contribute to both hypomethylation and DNA damage is also consistent with the existing correlation between the breakpoints in chromosomal rearrangements and DNA methylation patterns in breast cancer [174] and HGSC [175]. In breast cancer cells, chromosomal breakpoint intervals colocalize with differentially methylated regions [174]. For HGSC, global DNA hypomethylation (+) tumors had significantly higher levels of chromosomal instability than global DNA hypomethylation (−) tumors, and notably, CNVs were enriched in hypomethylated blocks [175]. The role of the G4 structure as a mediator of epigenetic modification was recently documented in another review [176,177].

**Figure 3:**
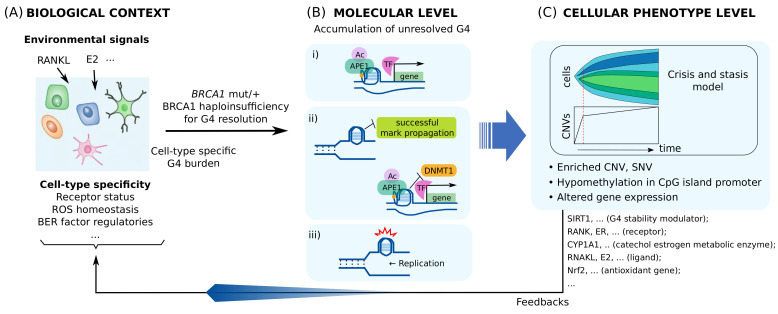
Model for tissue-specific tumorigenesis by BRCA1 deficiency. (**A**) How much of transcription regulatory G4/R-loop is generated at basal level is cell-type specific. (**B**) High burden on G4 processing causes multi-level molecular alterations by BRCA1 haploinsufficiency in the form of: (i) transcriptional alterations, (ii) epigenetic alterations, and (iii) genetic alterations. (**C**) These alterations contribute to phenotype evolution and modify the biological context of the cell by various factors, such as SIRT1, NRF2, estrogen receptor (ER)-E2 signaling, and RANK-RANKL signaling. Clones with the same pattern of copy number variations (CNVs) or single nucleotide variations (SNVs) expand as the tumor grows. One of the clonal expansion models for CNV, the Crisis and stasis model [178], is shown.

Multi-level alterations by BRCA1 haploinsufficiency for processing transcriptional regulatory G4s are illustrated in Figure 3. These alterations can be heterogeneous, depending on the insufficiency level and intra-cellular spatial distribution of BRCA1. This may give rise to varying feedback to G4/BER-mediated transcription, which may lead to additional heterogeneous phenotype evolution. Furthermore, some altered genes, such as *SIRT1*, may have their own functions associated with genomic instability and tumorigenesis, and may therefore contribute to the evolution of distinct phenotypes.

## 5. Discussion

G4 is a determinant in shaping the cell type-specific transcription and the mutational landscape of the cancer genome. The molecular mechanism of G4/BER-mediated transcriptional activation explains (i) the cell-type specificity of transcriptional regulatory G4s and (ii) the previously observed significant correlation between cell type-specific transcriptional activities and genome-wide G4 landscape and somatic CNVs. When certain transcriptional regulations that produces many G4s are activated in specific cells, the role of BRCA1 to resolve G4s becomes important. If the amount of G4 to be processed is greater than what can be processed by intracellular levels of BRCA1, genomic and epigenetic alterations occur by the resulting persistent G4s. This explains the cell-type specificity of BRCA1 haploinsufficiency seen in BRCA1 mutation carriers and provides important insights into BRCA1-deficient tumors.

This molecular mechanism of cell type-specific tumorigenesis by BRCA1 deficiency provides an integrated understanding of BRCA1-associated tumors. Until now, deficiency of HR-DSB repair has been the only considerable clinical factor in BRCAness tumors. However, as it is still correlated with HR-DSB repair deficiency, but not limited to this pathway, and addressing an underlying molecular mechanism, this integrated view may extend the possibilities of anticancer therapy or cancer prevention. Furthermore, the mechanism by which multi-level alterations are induced by defective G4 processing provides molecular insights into the nature of tumor clonal evolution. In the case of TNBC, it is known that CNV and aneuploid rearrangements remain stable after an early short burst of crisis, while point mutations evolve gradually, generating an extensive clonal diversity with a much higher mutation rate than ER+ tumor [179,180]. 

Further questions might emerge, such as whether this pathogenesis is valid for all BRCAness tumors, even in the absence of pathogenic alterations of BRCA1. There are reports that a marked increase in the needs for BRCA1 causes BRCAness tumor formation in normal BRCA1 carrier or in tissues other than in breast or ovary tissue. For instance, the ectopic expression or endogenous activation of heterochromatin-encoded satellite RNA phenocopied BRCA1-deficient cells and promoted breast cancer formation. This is associated with the insufficient function of BRCA1 by binding to satellite RNAs [181,182]. Ewing sarcoma, due to EWS-FLI1 fusion, also exhibits a phenotype similarity to BRCA1-deficient tumors [183]. Although Ewing sarcoma cells showed robust BRCA1 expression with no known mutations, HR was impaired in Ewing sarcoma. It was also demonstrated that this was highly associated with R-loop accumulation, and BRCA1 overexpression restored homologous recombination [183]. 

The recurring question of how CNVs are related to tumor progression, which has been studied primarily in terms of its impact to gene expression [184,185,186,187], can also be reconsidered. Rather than CNVs as a variation of gene dosage that changes the expression levels of genes with some degree of a genetic compensation [188], the correlation between altered gene expression and CNVs should be considered in the context of both originating from the same cause, unresolved G4s. Because G4 is a key element for transcriptional regulation, an altered transcriptional activity, implying misregulated G4s, is more likely to be responsible for CNVs in BRCAness tumors. Then in turn, this suggests considering how the generated CNVs change G4 structures and gene expression via a G4/BER-mediated mechanism. Given the crucial role of G4s in 3D genome organization through interactions with key architectural proteins [67,68,69], this is in line with recent views of analyzing CNVs in terms of rearrangement in which distant regions of the genome are brought together [189]. In recent years, many similar studies have been conducted with keywords such as enhancer-hijacking, TAD disruption, 3D genome rewiring, and insulator dysfunction [189,190,191,192].

In addition, because the RANK/RANKL pathway, that is, NF-κB activated transcription, in LP cells is a critical contributor to TNBC, an emerging question is whether BRCAness correlates with deficiency of R-loop/G4 processing and NF-κB activated transcription at the pan-cancer level. If not, the next question is which transcriptional activation, other than NF-κB activated transcription, causes high G4 levels. Additionally, how high frequencies of *TP53* mutation and G4/BER-mediated transcriptional activation are correlated needs to be elucidated. 

This cell type-specific tumorigenic mechanism of BRCA1 deficiency reflects the target-independent and molecular mechanism-based role of BRCA1 in transcriptional regulation. A similar concept was proposed for the oncoprotein MYC family [193]. In a recent review of the interactomes of MYC and MYCN, the function of MYC was addressed from new perspective, focusing on the mechanisms by which MYC proteins modulate transcription [193]. The function of MYC has been described as a stress resilience of basal transcription, including promoting transcription termination upon the stalling of RNA polymerase II (RNAPII) and coordinating transcription elongation with DNA replication and cell cycle progression. This may be an oncogenic ability of MYC, which promotes tumorigenesis independently of either global or specific changes in gene expression [193]. Incidentally, the target gene-independent functions of these two proteins, the MYC family and BRCA1, are interrelated. BRCAness tumors exhibit high levels of *MYC* amplification [5,6]. MYC is a neighboring node with BRCA1 in the genetic interactome map for high-level somatic CNVs, and one of the top genes with the largest copy number alteration [194]. Since 8-OxoG in the promoter and BER processes are essential for initiation of MYC-mediated transcription [104,105], MYC is likely to participate in the G4/BER-mediated mechanism. Moreover, when MYCN fails to release RNAPII from transcriptional pause sites, MYCN recruits BRCA1 to promoter-proximal RNAPII-stalled regions for transcriptional activation [195]. 

Returning to the discussion of the target-independent and molecular mechanism-based role of BRCA1, the tissue-specific tumor susceptibility of BRCA1 cannot be explained without considering the detailed molecular mechanisms of biological processes in which BRCA1 is involved and its molecular functions within these processes. The best-known biological processes in which BRCA1 is involved are DNA damage repair and replication fork protection [13,196]. Since G4s are obstacles in DNA replication that cause DNA damage, it was previously known that DNA helicases processing G4s are involved in these biological processes [197]. However, a direct association between G4s or DNA helicases and the function of BRCA1 was only recently reported. Recent studies have shown that BRCA1 is required for DNA damage repair in the form of a complex with helicases that resolve G4s and R-loops, such as senataxin (SETX) [198,199] or DHX9 [200,201]. These BRCA1-DHX9 or BRCA1-SETX complexes play an important role for DSBs occurring at transcriptionally active loci to be preferentially repaired by HR [199,201]. The molecular mechanism of DNA repair and replication restart in the presence of G4s or R-loops is very important for understanding cell-type-specific tumorigenesis by BRCA1 deficiency, but the details remain to be elucidated. 

In this paper, we reviewed the association of BRCA1 with transcriptional regulatory G4s, considering recent advances on the cancer genome and fundamental cellular processes on DNA such as transcription and DNA replication. Tissue-specific tumorigenesis by BRCA1 deficiency can be explained by cell type-specific levels of transcriptional regulatory G4s and the role of BRCA1 in resolving it. Comprehensive consideration of the consequences of BRCA1 deficiency in relation to G4s at multi-omics allows us for integrated understanding of individual reports about BRCAness tumors. This molecular mechanism of cell type-specific tumorigenesis by BRCA1 deficiency will provide new insights into BRCAness tumors, which take us a step further in the direction of developing novel therapeutic and preventive strategies.

## Data Availability

Not applicable.
